# Aging in Sensory and Motor Neurons Results in Learning Failure in *Aplysia californica*

**DOI:** 10.1371/journal.pone.0127056

**Published:** 2015-05-13

**Authors:** Andrew T. Kempsell, Lynne A. Fieber

**Affiliations:** University of Miami, Rosenstiel School of Marine and Atmospheric Science, Department of Marine Biology and Ecology, Miami, Florida, United States of America; Texas A&M University - Corpus Christi, UNITED STATES

## Abstract

The physiological and molecular mechanisms of age-related memory loss are complicated by the complexity of vertebrate nervous systems. This study takes advantage of a simple neural model to investigate nervous system aging, focusing on changes in learning and memory in the form of behavioral sensitization in vivo and synaptic facilitation in vitro. The effect of aging on the tail withdrawal reflex (TWR) was studied in *Aplysia californica* at maturity and late in the annual lifecycle. We found that short-term sensitization in TWR was absent in aged *Aplysia*. This implied that the neuronal machinery governing nonassociative learning was compromised during aging. Synaptic plasticity in the form of short-term facilitation between tail sensory and motor neurons decreased during aging whether the sensitizing stimulus was tail shock or the heterosynaptic modulator serotonin (5-HT). Together, these results suggest that the cellular mechanisms governing behavioral sensitization are compromised during aging, thereby nearly eliminating sensitization in aged *Aplysia*.

## Introduction

Brain aging is associated with the progressive decline of neurophysiological processes and an increased prevalence of memory impairments. Some loss of neurons occurs with age, but more relevant is a reduction in synaptic contact and the efficiency of synaptic transmission between these contacts with age. In mammals, the number of neuronal synapses and density of dendritic spines decrease during aging [1–4] resulting in weaker synapses that are less capable of short-term plasticity [5]. In the rodent hippocampus, aging is associated with a decrease in synapses in the dentate gyrus and area CA1, a decrease in NMDA-receptor-evoked responses at perforant path synapses onto dentate gyrus granule cells, and changes in Ca^2+^ regulation in area CA1 [6]. As these changes in synaptic structure and function accumulate during aging, cellular analogs of learning and memory, including long-term potentiation (LTP) and long-term depression (LTD), are disrupted. Hippocampal aging is associated with deficits in the induction and maintenance of LTP and lower thresholds for LTD [7]. Altered synaptic plasticity in aged hippocampal networks results in age-related memory loss including reduced performance in spatial memory tasks [8, 9].

Neural networks in mammalian models are vastly complex, making it challenging to connect behavioral aging with physiological aging. The marine snail *Aplysia californica* (*Aplysia*) provides a unique animal model to study the neurobiology of aging with the advantages of a simple nervous system and short, one-year lifespan. *Aplysia* is an opistobranch mollusk and a member of the large superclade of bilaterian animals, the Lophotrochozoa. While Ecdysozoans including *Drosophila* and *C*. *elegans* have undergone severe gene loss and sequence divergence [10], Lophotrochozoans do not have such derived genomes [11]. A lower amino acid replacement rate and thus a slower evolving genome when compared to the Ecdysozoans imply that *Aplysia* may have more genes in common with higher order species including humans.

One of the best mapped neuronal circuits in the *Aplysia* nervous system serves the tail withdrawal reflex (TWR). Direct stimulation to the tail initiates TWR, a monosynaptic reflex involving identified primary mechanosensory neurons (SNs) in the pleural ganglia and motoneurons (MNs) in the pedal ganglia [12, 13]. Sensitization in TWR is a simple form of nonassociative learning in which tail withdrawal becomes enhanced after harmful stimuli such as tail shock [14–16]. Three temporal phases of memory for sensitization can be induced depending on the amount and pattern of tail shocks [17]. A single tail shock induces short-term memory lasting ≤30 min, whereas repeated tail shocks induce intermediate-term memory lasting ≤90 min and long-term memory lasting ≥24 h. The neuromodulator serotonin (5-HT) is released by facilitatory interneurons onto the sensorimotor synapse during behavioral sensitization, resulting in increased tail SN excitability and heterosynaptic facilitation of tail SN-MN transmission [14, 18–23].

Three temporal forms of synaptic facilitation can be induced and the underlying mechanisms governing this proxy for synaptic plasticity are well-studied [24, 25]. In short-term facilitation (≤30 min), a brief pulse of 5-HT activates adenylyl cyclase in presynaptic neurons, causing an increase in cyclic adenosine monophosphate (cAMP) and activation of protein kinase A (PKA). PKA mediates short-term facilitation by covalent modifications of channel activation, resulting in altered channel activity including closure of K+ channels. Reduced K+ current increases action potential (AP) duration and thus raises the amount of presynaptic cytosolic Ca^2+^. Ca^2+^ influx activates Ca^2+^/calmodulin-dependent protein kinase II, which then phosphorylates synapsin I. Phosphoylated synapsin I loses its affinity for synaptic vesicles, releasing them from the cytoskeleton and leading to increased synaptic release and temporary synaptic strengthening. Longer exposures to 5-HT cause the prolonged activation of PKA as well as activation of protein kinase C (PKC), inducing intermediate-term facilitation (≤90 min) and long-term facilitation (≥24 h). Intermediate-term facilitation requires translation while long-term facilitation requires both transcription and translation. Prolonged PKA activity results in the phosphorylation of transcription factors such as CREB-1, stimulating RNA and protein synthesis.

This study investigated whether behavioral sensitization in TWR undergoes age-related declines that involve changes in synaptic facilitation between SNs and MNs known to be involved in the reflex circuit. We measured short-term memory for sensitization in TWR in identified animals as they aged. We also measured short-term changes in tail SN excitability and synaptic facilitation of tail SN-MN transmission. The results indicate that aging of the neural circuit for TWR resulted in learning failure, including the ability to sensitize TWR in intact animals, and related impairment of synaptic facilitation of the SN-MN circuit underlying TWR.

## Materials and Methods

Cohorts of *Aplysia* from the University of Miami National Resource for *Aplysia* were reared from egg masses of wild-caught animals and were either full or half siblings. Animals were fed an ad-libitum diet consisting of *Gracilaria ferox* and *Agardhiella subulata* and reared as described previously [26]. Four hatchery-reared animals of different weights were assigned to each cage to allow for identification of individual animals by monthly weight measurements. Sexual maturity for a cohort of animals was designated as the day the first egg mass was laid.

Behavioral and electrophysiological experiments were done at two points in the adult life of this annual animal: mature and aged II, as described in Kempsell and Fieber (2014) [27]. Mature animals were age 7–8 mos and had reached sexually maturity <1 month earlier. Aged II animals were age 12–13 mos, designated as advanced age animals. They had significantly reduced performance in the righting reflex, TWR, and biting response compared to mature and aged I siblings, as described in the previous paper [27].

### Sensitization of TWR in freely behaving Aplysia

Animals were placed individually in 48 x 27 x 20 cm translucent plastic cages filled with 20–21°C seawater to a depth of 15 cm and allowed to acclimate for 5 min prior to measurements. A trained experimenter who was unaware of the age of the animals conducted behavioral measurements with the help of an assistant. The experimenter measured TWR amplitude and duration while the assistant recorded each measurement. We opted to score responses from live observations. Measuring TWR amplitude and duration from video recordings was not done due to difficulties in establishing a camera angle that properly recorded all animals.

**Fig 1:**
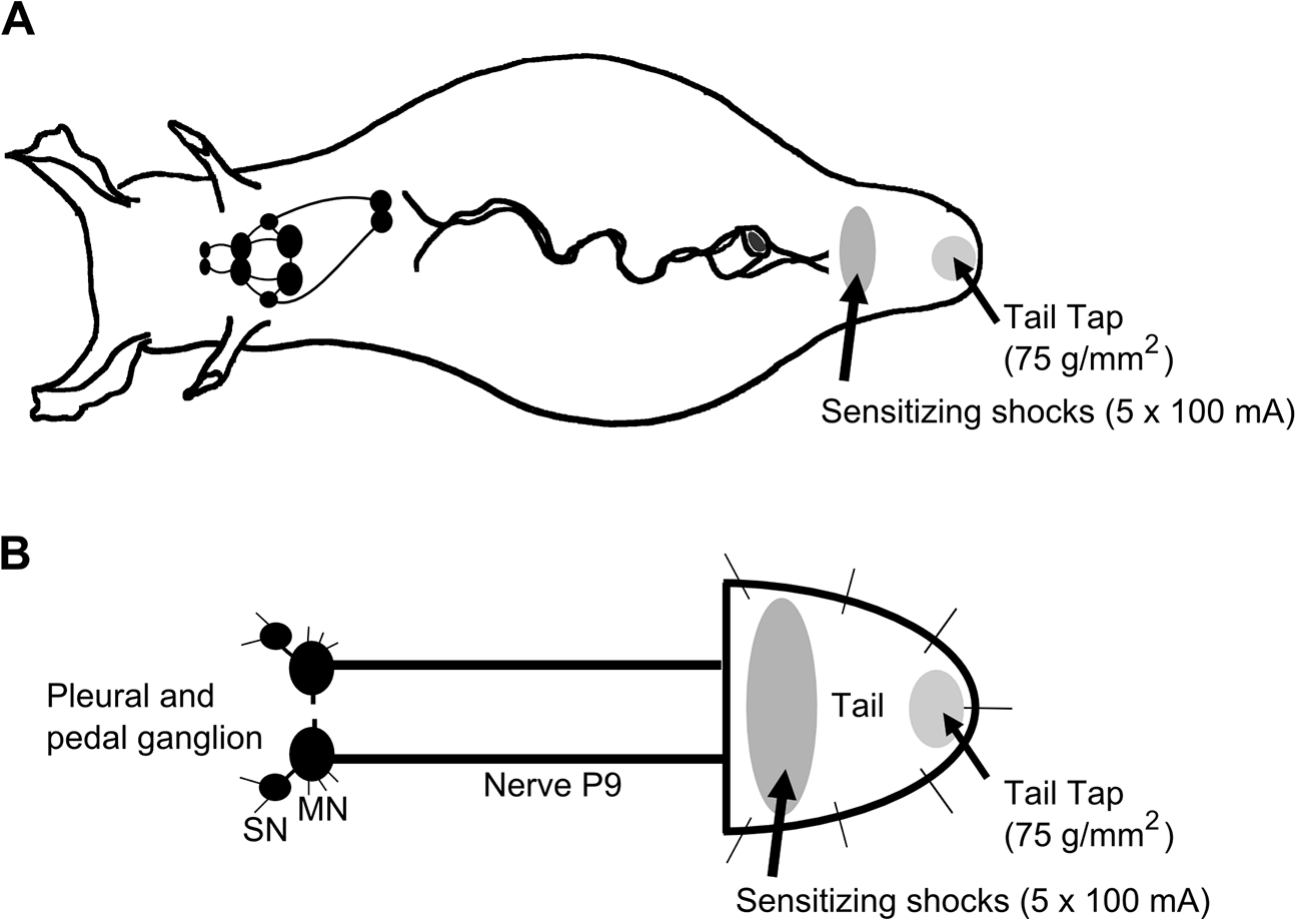
Schematic diagram of *Aplysia* illustrating regions of the tail subject to mechanical test stimulation and sensitizing electrical shocks. A) Behavioral experiments were performed in the intact animal. TWR amplitude and TWR duration were monitored in response to tail tap (75 g/mm^2^). After 3 baseline pretest taps, each animal received sensitization training consisting of five 1.5 sec, 100 mA shocks delivered to a site immediately posterior to the parapodial convergence. TWR amplitude and TWR duration in response to test tail tap were recorded 15, 30, 45, and 60 min after training and compared to baseline. B) Electrophysiological experiments consisted of a reduced preparation that involved the pleural-pedal ganglia, nerve P9, and attached tail. Tail SN and MN responses to mechanical tail tap were assessed before and after sensitization training as described in A.

The same 18 animals were measured for sensitization in TWR at mature and aged II time points following previously defined protocols [16, 27]. An animal was placed on its foot in the center of the cage and allowed to acclimate for 5 min. The animal’s resting body length was measured with a ruler. A 500 ms tap to the tip of the tail at a stimulus pressure of 75 grams/mm^2^ caused tail withdrawal towards the center of the body and signified the start of the reflex. The retracted body length was then measured. The time to relax the tail to ~30% of original tail length was recorded, and signified the end of the reflex and the reflex duration. TWR amplitude was calculated as the fraction of starting body length withdrawn following tail touch. TWR amplitude and TWR duration were measured at 15, 10 and 5 min before sensitizing electrical shocks to the tail (-15, -10, -5 min) and the average of these 3 measurements was designated as baseline. Five minutes after the last baseline tail tap, sensitizing tail shocks were then delivered, consisting of five 1.5 sec, 100 mA electrical shocks, with an interstimulus interval of 1 sec, delivered to the tail immediately posterior to the parapodial convergence at a different site than that used to measure baseline TWR (Fig 1A). This site was ~2–3 cm anterior to the tip of the tail. Next, TWR was again elicited by tail tap 5, 15, 30, and 60 min following sensitizing shocks, and amplitude and duration were measured.

### General Intracellular recording methods

Glass capillary microelectrodes of 5–15 MΩ resistance were used for intracellular recordings in tail SNs and MNs. All recordings were made at room temperature of 21–23°C using pClamp10 software with BRAMP-01R and ELC-01MX amplifiers (ALA Scientific Instruments, Farmingdale, NY) connected to a PC and Digidata 1440A A/D converter.

Behavioral experiments were conducted at 20–21°C while electrophysiological experiments were conducted at room temperature (21–23°C).

### Intracellular recording of TWR in semi-intact tail preparation

In all electrophysiological experiments, a ganglia-tail preparation was used that consisted of the left or right pleural-pedal hemiganglia connected to the tail by nerve p9 [28], with all other connectives severed (Fig 1B). A preparation that included the head ganglia produced no differences in tail SN and MN responses compared to the more reduced preparation that isolated SN and MN innervation to the pleural and pedal ganglion. Animals were anesthetized by injection of isotonic MgCl_2_ (~50% animal weight by volume) into the body cavity. Pleural-pedal ganglia, nerve p9, and attached tail were removed and pinned tightly to a Sylgarded dish. The other ganglia were removed from the remaining tissue to euthanize the animal, and unneeded ganglia and tissue were discarded. The pins were positioned in the reduced tail preparation to minimize tail contraction following mechanical and electrical stimulation. The protective sheath surrounding the ganglia was removed mechanically. Ganglia were maintained for the duration of the experiment in artificial seawater (ASW) via a gravity-fed perfusion pipette located ~5 mm from the ganglia. Tail SNs and MNs were identified according to previously defined methods [12–14]. Tail SNs of the ventral caudal region of the pleural ganglia (PVC) were not spontaneously active and had resting potentials of -40–-55 mV, but when receptive fields on the tail were stimulated, produced AP of 60–100 mV amplitude [12]. Tail pedal motoneurons P5-7 were spontaneously active with resting potentials of -40–-70 mV and exhibited increased AP firing in response to mechanical stimulation of the tail [14].

Electrophysiological experiments involved tail SN and MN responses to injected current or mechanical tail stimulation before and after sensitizing stimuli. Sensitizing stimuli consisted of tail shocks (described above), or application of 5-HT. In all electrophysiological experiments, three pre-treatment (-shock or -5-HT) tests were recorded with a 5 min interval between tests, and the baseline response was calculated from their average. Post-treatment test responses were then evoked at 5, 15, and 30 min after one of these treatments: tail shocks, or 5-HT application for 10 min from the perfusion pipette. Tail shocks were delivered as in freely behaving animals to a site on the tail immediately posterior to the parapodia, ipsilateral to the ganglion under investigation, at a position that did not include the mechanoreceptive field of the tail SN. In electrophysiological experiments investigating heterosynaptic modulation by 5-HT, 5-HT (20 μM) was perfused onto the pleural-pedal ganglion for 10 min. This concentration was previously found to induce forms of modulation including synaptic facilitation, increased AP duration and increased excitability in tail SNs [29, 30]. Tail motoneurons were held at -90 mV to arrest AP generation.

To measure excitability in tail PVC SNs, a protocol was modified from previous experiments [31, 32]. Depolarizing current was applied to PVC SNs in increasing increments of 0.1 nA for 500 ms until the neuron fired a single AP, then the single AP evoked by this depolarization was confirmed 3 times (baseline response; 5 min interval between tests). After treatments of either sensitizing tail shocks or 5-HT, the same depolarizing current as in baseline was applied, and the number of AP fired by the SN was counted.

To assess monosynaptic transmission between tail SNs and MNs, synaptic facilitation was evaluated by measuring the amplitude of a single SN-evoked excitatory postsynaptic potential (EPSP) in the MN and comparing EPSP amplitude to baseline. A single AP was evoked in the tail SN by 50 ms depolarizing current injection and the amplitude of the resulting EPSP was measured at time points corresponding to 15, 10 and 5 min before and 5, 15, and 30 min after the end of sensitizing tail shocks or 5-HT treatment and compared. EPSP latency was defined as the time from tail SN AP initiation to tail MN EPSP initiation. No homosynaptic depression was observed with the interstimulus intervals used in these experiments.

To assess synaptic transmission between tail SNs and MNs during mechanical tail tap, protocols were modified from previous experiments [14, 28]. The responses to a single 500 ms tail tap at a stimulus pressure of 75 grams/mm^2^ at -15, -10, and -5 (before), and 5, 15, and 30 min following sensitizing tail shocks were compared in mature and aged II tail SNs and MNs. Tail tap evoked AP in tail SNs and complex EPSP in tail MNs. The number of AP fired in response to tail tap was compared in mature and old tail SNs. For analysis of complex EPSP in tail MNs, the amplitude of the first evoked EPSP was averaged in mature and in aged II MNs and compared.

In electrophysiological experiments investigating modulation of tail SN biophysical properties by 5-HT, resting membrane potential was monitored while 5-HT (20 μM) in ASW was perfused onto the pleural-pedal ganglion. After 3 min 5-HT perfusion, a single AP was evoked in tail SNs by 3 ms depolarizing intracellular current injection. Repolarization amplitude, afterhyperpolarization (AHP) amplitude, and AP duration were compared before and after 5-HT treatment in mature and aged II tail SNs.

### Solutions

Extracellular solution consisted of ASW containing (mM) 417 NaCl, 10 KCl, 10 CaCl_2_, 55 MgCl_2_, and 15 HEPES-NaOH, pH 7.6. The pipette solution for intracellular recordings in intact ganglia consisted of 3 M KCl. Solutions containing 20 μM 5-HT in ASW were prepared daily from 0.5 M stocks as previously reported [33]. All reagents were from Sigma-Aldrich (St. Louis, MO, USA).

### Data Acquisition and Analysis

Data were expressed as mean ± SE. For behavioral experiments, significant differences from baseline were assessed via one-way within subjects (repeated-measures) ANOVA with Tukey’s posthoc test, as follows. After the ANOVA, means at individual time points were compared against baseline mean using Tukey’s posthoc test to determine significant sensitization at that time point. For electrophysiological experiments, significant differences to determine facilitation of averaged neuron responses were assessed via one-way within subjects ANOVA with Tukey’s posthoc test. Baseline responses were compared to responses after treatments of sensitizing tail shocks or 5-HT. To compare mature and aged II responses, 2-sample t-tests were used. All analyses were performed using the open source R statistical program (R Foundation for Statistical Computing, Vienna, Austria). P-values are all 2-tailed. Differences at p≤0.05 were accepted as significant.

## Results

Sexual maturity occurred by age 7 mos and median lifespans were 12.9 and 12.7 mos for the 2 cohorts studied. The percentage of animals that died before the aged II time point was 8.3% and 6.3% respectively. Morphological and aging characteristics of these cohorts were reported in a previous study [27]. Briefly, growth was steady and peaked shortly after first sexual maturity and maximal egg production. A significant reduction in body mass was observed from age 11–13 mos in these cohorts (p≤0.05, 2-sample t-tests), however, no difference in body mass was observed between stages mature and aged II.

### Sensitizing shocks in intact animals increased TWR in mature but not aged II *Aplysia*

**Fig 2:**
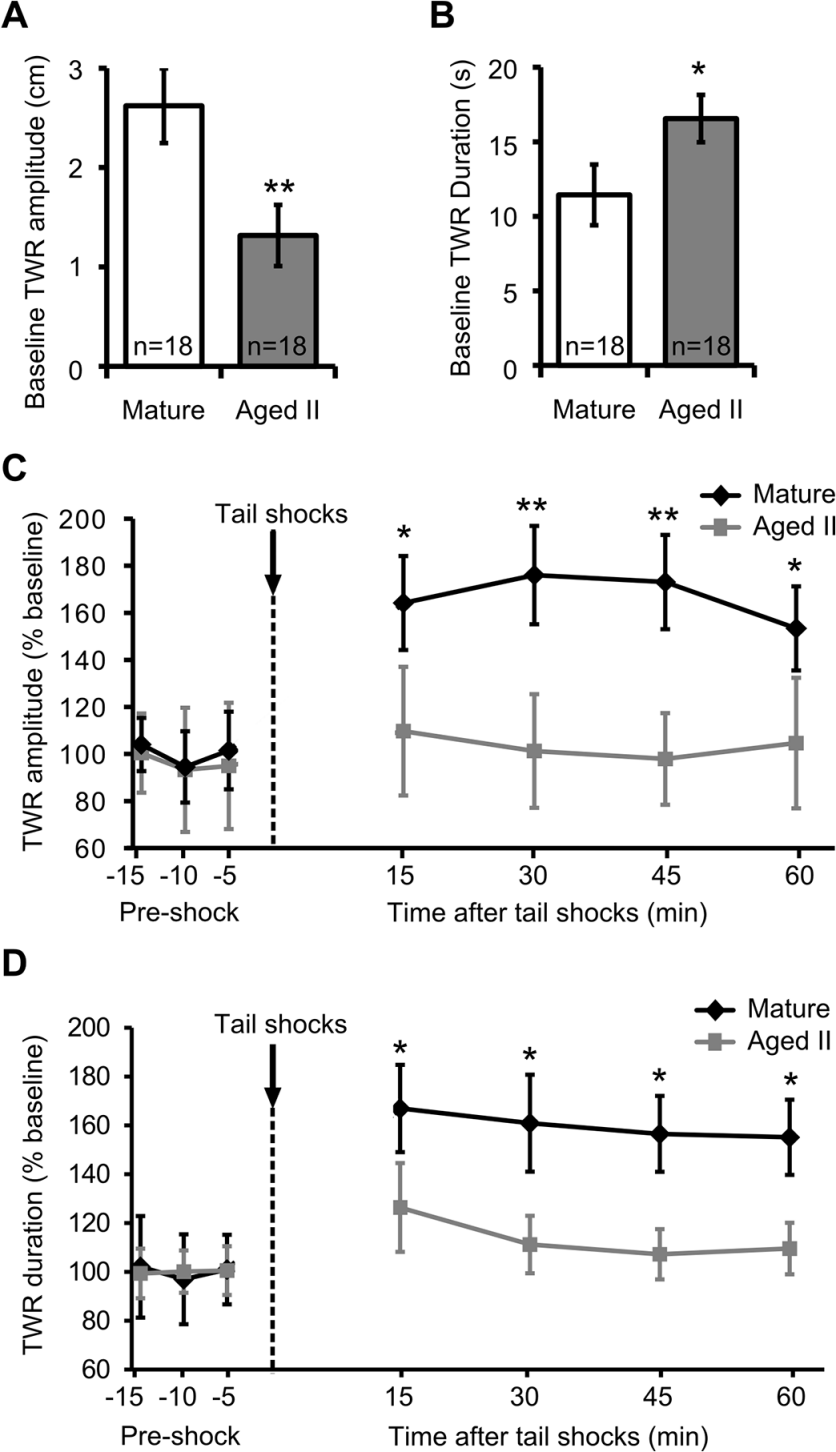
Sensitization in TWR of intact animals declined during aging. A) TWR amplitude in baseline pretests decreased, while (B) TWR duration increased significantly during aging. * and ** denote significant difference compared to mature at p≤0.05 and p≤0.01, respectively, via 2-sample t-tests. C) TWR amplitude and (D) TWR duration increased significantly in mature animals following five sensitizing shocks (1.5 s each, 100 mA, AC, 1 s interstimulus interval) delivered to the anterior tail, while in the same animals at stage aged II no shock-induced change in amplitude or duration was observed. * and ** denote increase compared to baseline at p≤0.05 and p≤0.01, respectively, via Tukey’s posthoc tests following repeated measures ANOVA (n = 18).

Baseline TWR amplitude in 18 sibling animals was significantly weaker (Fig 2A, p≤0.01, 2-sample t-test) and TWR duration was significantly slower (Fig 2B, p≤0.05, 2-sample t-test) when the animals reached aged II compared to their performance when mature, as noted previously [27]. Prior work in *Aplysia* has demonstrated that sensitizing tail stimuli increased TWR amplitude and duration [14–16]. To investigate if short-term memory for sensitization in TWR was disrupted during aging, the amplitude and duration of tail withdrawal in response to tail tap were measured before and after 5 sensitizing shocks (1.5 s each, 100 mA, AC, 1 s interstimulus interval). No change in response amplitude or duration was found at a 5 min interval between baseline tail taps (Fig 2C and 2D). In mature animals, five sensitizing shocks delivered to the anterior tail resulted in a significant increase in mean TWR amplitude at 15, 30, 45, and 60 min post-shock (Fig 2C; p≤0.05 at 15 and 60 min time points compared to baseline, p≤0.01 at 30 and 45 min time points; Tukey’s posthoc analysis). TWR duration also increased significantly in mature animals after sensitizing shocks (Fig 2D; p≤0.05 at each time point compared to baseline; Tukey’s posthoc analysis). Shocks did not significantly elevate TWR amplitude (Fig 2C) or TWR duration (Fig 2D) in aged II *Aplysia* compared to baseline. Thus, short forms (≤60 min) of memory for sensitization in TWR were absent in aged II *Aplysia*.

Between group differences in TWR amplitude and duration also were significant following sensitizing tail shocks. TWR amplitude and duration measured at each of 15–60 min following sensitizing shocks were significantly greater in mature compared to aged II animals (p≤0.05; Tukey’s posthoc analysis).

### Sensitizing shocks in reduced ganglia-tail preparations increased excitability in mature but not aged II tail SNs

Mean resting potential and mean input resistance did not change during aging in either tail SNs (resting membrane potential: -45.8±4.2 mV for mature, -44.9±6.2 mV for aged II; input resistance: 26.1±5.1MΩ for mature, 25.3±4.6 MΩ for aged II; p>0.05 in each case; 2-sample t-test) or tail MNs (resting membrane potential: -54.3±5.3 mV for mature, -53.2±10.4 mV for aged II; input resistance: 10.5±2.1 MΩ for mature, 9.9±2.7 MΩ for aged II). These values were consistent with those previously reported at ages mature and aged II [27].

**Fig 3:**
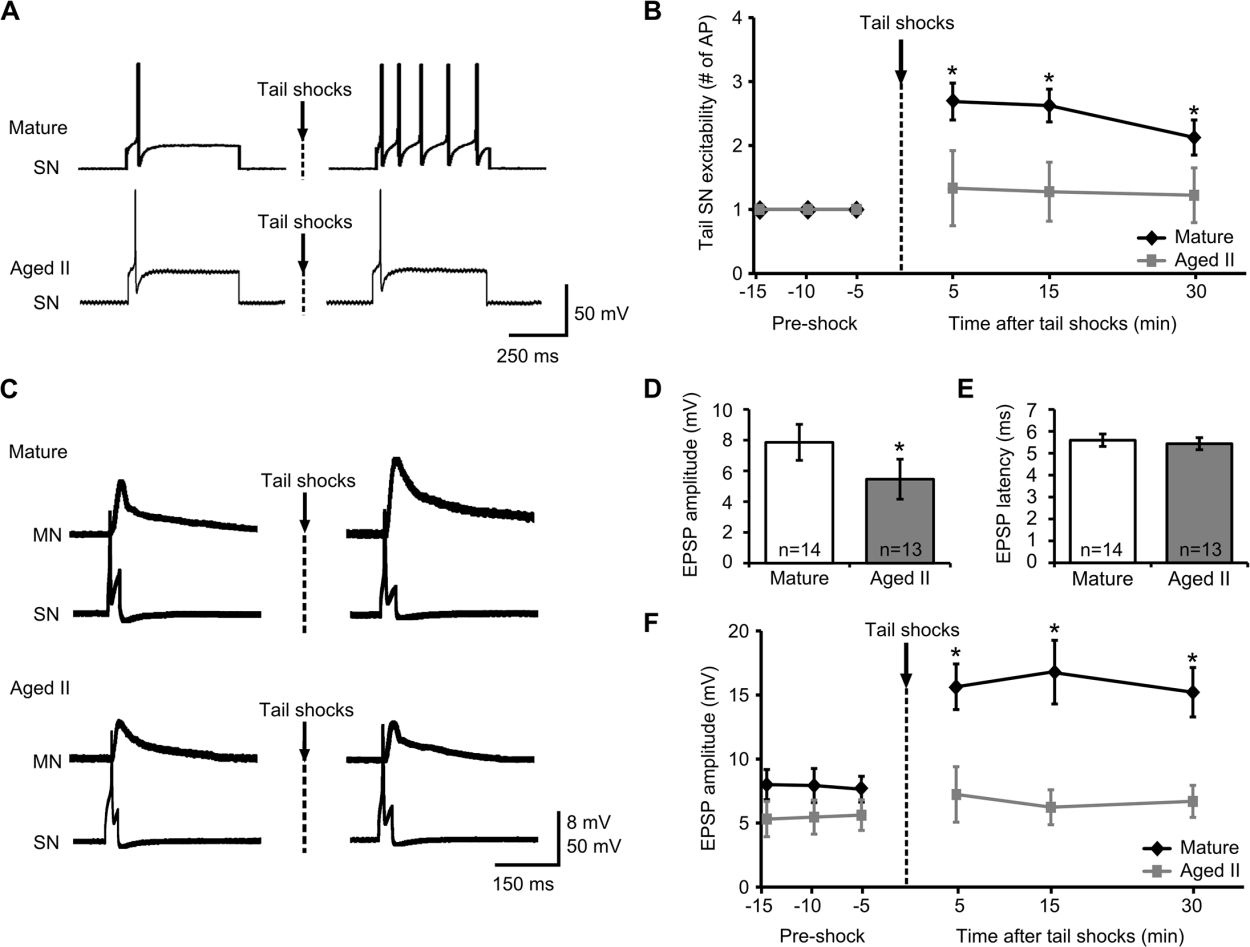
Synaptic facilitation between tail SNs and MNs following sensitizing tail shocks declined during aging. A) Representative responses during depolarizing current injection in tail SNs before and 5 min after sensitizing tail shocks in mature and aged II animals. B) In mature tail SNs, the same depolarizing current injection that yielded a single AP in baseline conditions evoked significantly more AP 5, 15, and 30 min after sensitizing tail shocks. * denotes significant increase compared to baseline at p≤0.05, Tukey’s posthoc tests (n = 16). No change in AP number was observed following sensitizing tail shocks in aged II tail SNs (n = 20). C) Monosynaptic facilitation between tail SNs and MNs following sensitizing tail shocks decreased during aging. A single AP evoked in tail SNs by 50 ms injected current resulted in a monosynaptic EPSP in tail MNs in mature (n = 14) and aged II (n = 13) preparations. Tail MNs were hyperpolarized to -90 mV. D) Baseline EPSP amplitude was significantly decreased in MNs of aged II preparations. * denotes significant decrease compared to mature at p≤0.05, 2-sample t-test. E) Baseline EPSP latency did not change during aging. F) Following sensitizing tail shocks, EPSP amplitude increased significantly in mature but not in aged II tail MNs. * denotes significant increase compared to baseline at p≤0.05, Tukey’s posthoc tests.

Sensitizing shock has been shown to increase excitability in tail SNs [14, 34–36]. To determine whether aging affects this phenomenon, excitability of individual SNs was evaluated in mature and aged II reduced tail preparations before and after 5 rapidly applied sensitizing shocks (Fig 3; 1 sec interstimulus interval). In preparations from mature animals, the same depolarizing test pulse that elicited a single AP in each control tail SN elicited multiple AP in the SN after sensitizing shocks (Fig 3A and 3B; p≤0.05 at each time point compared to baseline; Tukey’s posthoc analysis). In contrast, no change in tail SN excitability was found following sensitizing shocks in preparations from aged II animals.

Between group differences in tail SN excitability following sensitizing shocks also were significant following sensitizing tail shocks (p≤0.05; Tukey’s posthoc analysis).

### Sensitizing shocks facilitated mature but not aged II tail SN-MN synapses

Previous studies showed that monosynaptic EPSP amplitude increased following sensitizing stimulation in tail MNs [14, 37]. Monosynaptic EPSP were evoked in tail MNs by applying depolarizing current sufficient to initiate a single AP in tail SNs (Fig 3C). Baseline EPSP amplitude was significantly smaller in each MN studied from aged II preparations compared to mature (Fig 3D; p≤0.05; 2-sample t-test). The latency between AP initiation in tail SNs and evoked EPSP in tail MNs did not change during aging (Fig 3E). EPSP amplitude increased significantly in mature preparations at all time points after sensitizing shocks compared to pre-shock values (Fig 3F, p≤0.05 at each time point compared to baseline; Tukey’s posthoc analysis), whereas in aged II preparations no change was noted in EPSP amplitude after sensitizing shocks. EPSP amplitude also was significantly increased in mature animals following sensitizing shocks compared to aged II animals (p≤0.05; Tukey’s posthoc analysis). Thus, short-term facilitation (≤30 min) of tail SN-MN transmission induced by shocks was absent in aged II *Aplysia*.

### Sensitizing shocks increased the response to tail tap in mature but not aged II SNs and MNs

**Fig 4:**
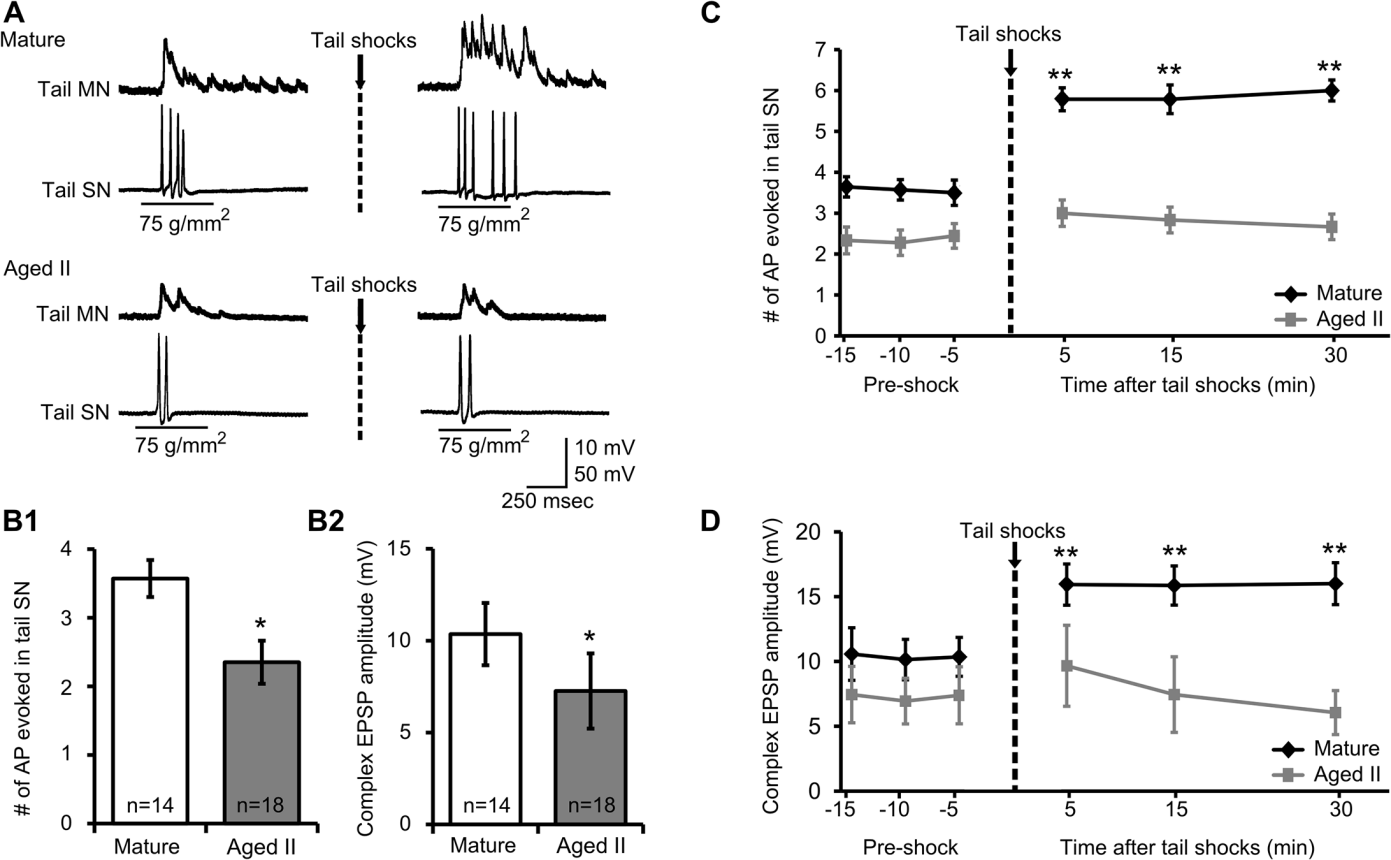
Tail tap-evoked responses of tail SNs and MNs decreased during aging. A) A single 500 ms tap to the tail at a stimulus pressure of 75 g/mm^2^ evoked AP generation in tail SNs and complex EPSPs in tail MNs from mature and aged II reduced tail preparations. B) The number of AP in response to baseline tail tap in tail SNs (B1) as well as the complex EPSP amplitude elicited in MNs (B2) decreased significantly during aging. * denotes significant decrease compared to mature at p≤0.05, 2-sample t-test. C) After sensitizing tail shocks, the number of AP in response to tail tap in tail SNs as well as (D) the complex EPSP amplitude in MNs increased significantly in mature but not in aged II preparations. ** denotes significant increase compared to baseline at p≤0.01, Tukey’s posthoc tests (n = 14 for mature, n = 18 for aged II).

The excitatory response of tail SNs and MNs to weak tail mechanical stimulation has been shown to increase after sensitizing shock [14, 28]. To further investigate changes in synaptic facilitation during aging, tail SNs and MNs were monitored during mechanical tail tap before and after sensitizing shocks in mature and aged II preparations. The sites for shocks and tail taps were separated (Fig 1B) to reduce activation of subpopulations of tail SNs with overlapping receptive fields [14, 17]. Tail tap evoked AP in individual tail SNs and complex EPSP in individual tail MNs (Fig 4A). In response to baseline tail tap, the number of AP fired in tail SNs and complex EPSP amplitude in tail MNs were significantly different between mature and aged II preparations, with aged II SNs exhibiting reduced AP firing (Fig 4B1) and aged II MNs exhibiting reduced complex EPSP amplitude (Fig 4B2; p≤0.05 in each case; 2-sample t-tests). Aging affected the response to sensitizing shocks as well. Whereas sensitizing shocks increased the responses of mature SNs and MNs to tail tap (Fig 4C and 4D; p≤0.01 at each time point compared to baseline; Tukey’s posthoc analysis), SNs and MNs from aged II preparations were unaffected. Furthermore, SN and MN responses were significantly greater in mature animals following sensitizing shocks compared to aged II animals (p≤0.01 at each mature time point compared to aged II time point; Tukey’s posthoc analysis). Thus, while tail SN and MN responses to mechanical tail tap were facilitated after sensitizing shocks in mature animals, responses from aged II animals were not.

### 5-HT treatment increased excitability in tail SNs of mature but not aged II *Aplysia*

**Fig 5:**
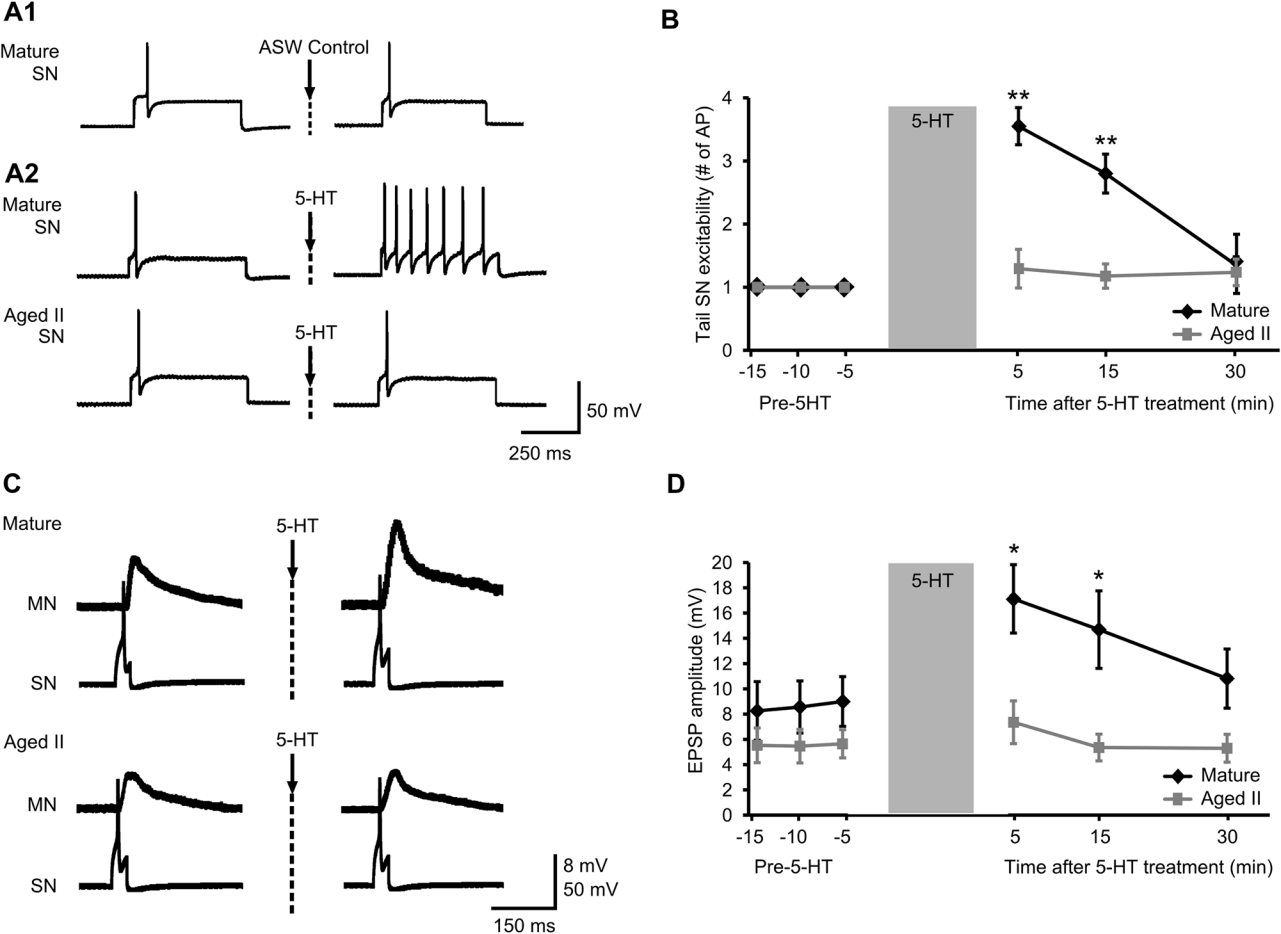
Synaptic facilitation between tail SNs and MNs following 5-HT treatment declined during aging. A) Representative responses during 500 ms depolarizing current injection in tail SNs before and 5 min after conclusion of 10 min perfusion of vehicle (ASW; A1) or 5-HT (20 μM; A2) onto the pleural-pedal ganglion. B) In mature tail SNs, the same depolarizing pulse that produced a single AP during baseline conditions evoked significantly more AP when assessed 5 and 15 min after 5-HT treatment. ** denotes significant increase compared to baseline at p≤0.01, Tukey’s posthoc tests (n = 16). No change in AP number was observed in aged II tail SNs following 5-HT treatment (n = 17). C) Monosynaptic facilitation between tail SNs and MNs following 5-HT treatment decreased during aging. A single AP evoked in tail SNs by 50 ms injected current resulted in a monosynaptic EPSP in tail MNs. D) Following 5-HT treatment, tail MN EPSP amplitude increased significantly in mature but not in aged II preparations. * denotes significant increase compared to baseline at p≤0.05, Tukey’s posthoc tests (n = 20 for mature, n = 17 for aged II).

Application of the heterosynaptic neuromodulator 5-HT to tail SNs has been shown to increase tail SN excitability and facilitate tail SN-MN synaptic transmission [14]. We examined 5-HT-induced changes in tail SN excitability during aging. No change in excitability was found in tail SNs (Fig 5A1) following 10 min treatment with vehicle (ASW control). In mature tail SNs, the same depolarizing pulse that yielded a single AP during baseline conditions evoked a significantly greater number of AP when excitability was assessed 5 and 15 min after 5-HT treatment (Fig 5A2 and 5B; p≤0.01 at 5 and 15 min time points compared to baseline; Tukey’s posthoc analysis). In aged II tail SNs, in contrast, no change in excitability was observed following 5-HT treatment. Tail SN excitability was significantly greater in mature animals when excitability was assessed 5 and 15 min after 5-HT treatment compared to aged II animals (p≤0.01 at 5 and 15 min; Tukey’s posthoc analysis).

### 5-HT facilitated mature but not aged II tail SN-MN synapses

Short-term synaptic facilitation induced by 5-HT applied to tail SN-MN synapses has been widely used as a cellular model of short-term memory for sensitization. Monosynaptic EPSP were evoked in tail MNs by applying depolarizing current sufficient to initiate a single AP in each tail SN studied (Fig 5C). After 10 min 5-HT perfusion of the pleural-pedal ganglion, MN EPSP amplitude increased significantly in mature (Fig 5D; p≤0.05 at 5 and 15 min time points compared to baseline; Tukey’s posthoc analysis) but not in aged II preparations. EPSP amplitude was significantly greater in mature animals after 5-HT treatment compared to aged II animals (p≤0.05 at 5 and 15 min in mature compared to aged II; Tukey’s posthoc analysis). Thus, short-term facilitation (≤30 min) of tail SN-MN transmission after 5-HT treatment was absent in aged II *Aplysia*.

### 5-HT altered biophysical properties of mature but not aged II tail SNs

**Fig 6:**
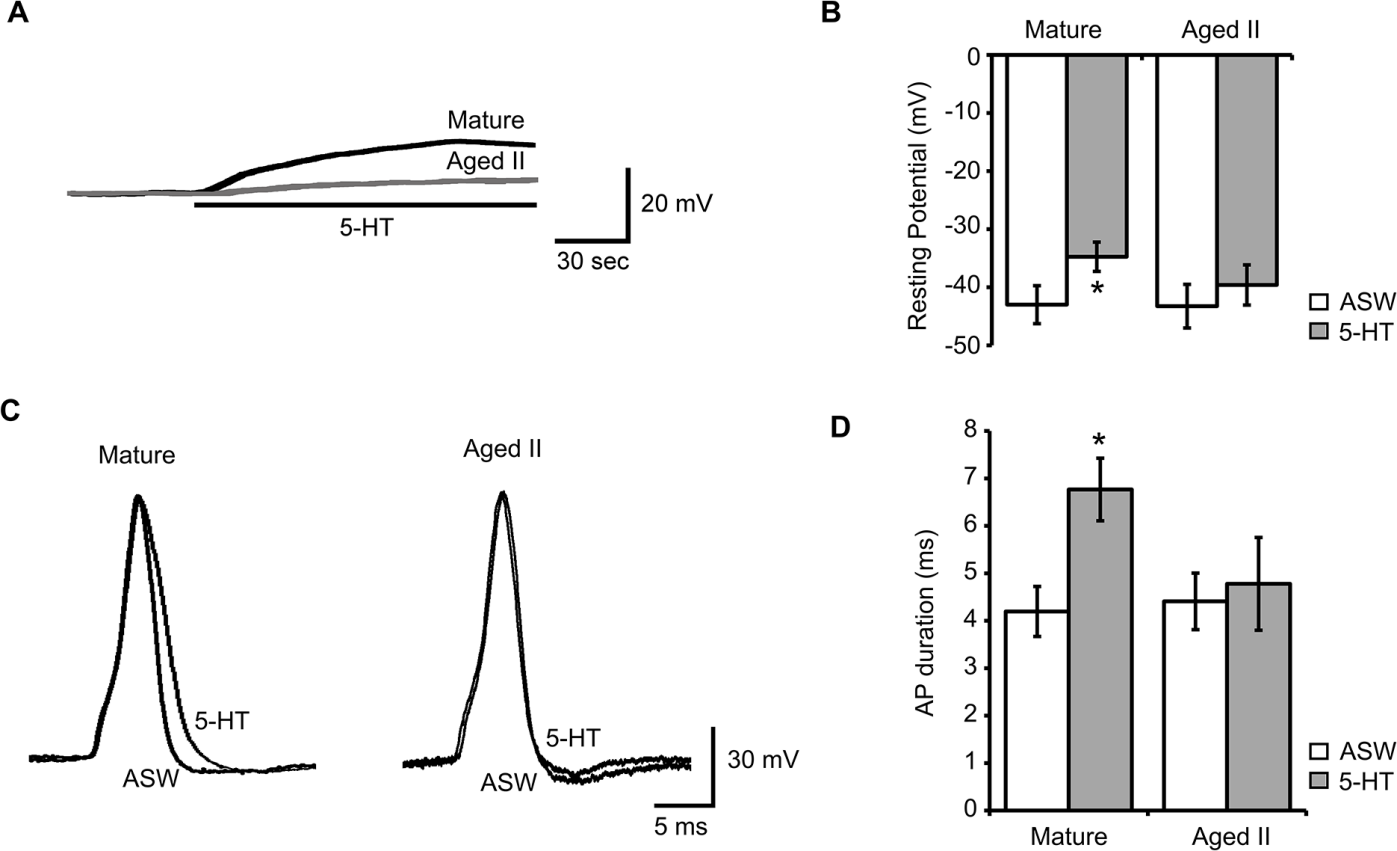
5-HT caused increased excitability in SNs of mature but not aged II *Aplysia*. A) Membrane potential in SNs in response to 5-HT (20 μM) perfusion onto the pleural-pedal ganglion. B) 3 min of 5-HT significantly depolarized mature but not aged II tail SNs. * denotes significant difference from ASW control at p≤0.05 via paired t-test. C) Injection of 3 ms depolarizing current evoked a single AP in tail SNs that was compared before (ASW) and after 5-HT treatment. D) AP duration increased significantly in mature but not aged II tail SNs after 3 min 5-HT. * denotes significant difference from ASW control at p≤0.05, paired t-test (n = 25 for mature, n = 23 for aged II).

5-HT is an important neuromodulator implicated in forms of learning and memory including behavioral sensitization in TWR. Previous studies showed that 5-HT treatment induces changes in biophysical properties of tail SNs that enhance excitability, including resting membrane potential and duration of evoked AP [34, 38]. In mature tail SNs, 5-HT treatment resulted in a slow and maintained depolarization of resting membrane potential, significantly different at 3 min in 5-HT from the initial measurement (Fig 6A and 6B; p≤0.05; paired t-test). In aged II tail SNs, the effects of 5-HT treatment were much more variable. No significant changes in resting membrane potential were observed in aged II tail SNs after 5-HT (Fig 6A and 6B).

Another hallmark of 5-HT induced facilitation of excitability in tail SNs was increased AP duration [38]. A single AP was evoked by 3 ms depolarizing intracellular current injection in tail SNs before and after 3 min 5-HT treatment (Fig 6C). Depolarization amplitude, afterhyperpolarization (AHP) amplitude, and AP duration were measured. In ASW before 5-HT treatment, AP duration (mature: 3.8±0.6 ms, aged II: 4.0±0.9 ms) and depolarization amplitude (mature: 88±11 mV, aged II: 82±13 mV) were not different between mature and aged II (Fig 6C and 6D). AHP amplitude (mature: 4.2±0.7 mV, aged II: 4.7±0.9 mV) also was not different, consistent with studies in other aging models that found no change in fast components of AHP amplitude during aging [39].

In mature tail SNs, 5-HT caused a significant increase in AP duration of 2.0±0.9 ms compared to ASW controls (Fig 6D; p≤0.05, paired t-test), but not in aged II animals. Thus changes in biophysical properties of tail SNs induced by 5-HT that facilitate excitability were absent in aged II *Aplysia*.

## Discussion

The experiments described here took advantage of a simple neural model to investigate nervous system aging, focusing on learning-induced changes that accompany behavioral sensitization in vivo and synaptic facilitation in vitro. These experiments were performed in cohorts of half sibling animals. The consistency of the results demonstrates the promise for aging studies of carefully reared *Aplysia*. Short-term memory for sensitization in TWR and short-term facilitation of the tail SN-MN synapse were examined during aging. The findings suggest that aging of the neural circuit for TWR resulted in learning failure, including the ability to sensitize TWR in intact animals, and related impairment of synaptic facilitation of the SN-MN circuit underlying TWR.

Baseline TWR declined in amplitude and slowed in duration during aging, and appeared to have related neural correlates. Tail SN excitability and tail MN EPSP amplitude decreased during aging, suggesting that aging negatively affects tail SN-MN synaptic transmission. Previously we noted that current injection to aged MNs did not change either the number of AP elicited, nor the threshold of aged MNs to fire AP [27]. The earlier results, combined with those here, suggest that aged MNs could be experimentally driven to perform similar to mature MNs, but that some aspects of their intrinsic excitability, such as EPSP amplitude, declined in aging. These results add to the growing relevance of *Aplysia* as a model with characteristic and measureable declines in defined neural circuits with age [27, 40–44].

We also found that short-term synaptic facilitation between tail SN-MN declined during aging whether the facilitating agent was tail shock or 5-HT, suggesting deficits in the underlying circuit for sensitization in TWR. Diagnostically, sensitizing shocks failed to increase excitability in aged II tail SNs or to increase the efficacy between tail SN-MN synapses, as occurred in mature preparations. Furthermore, treatment with the neuromodulator 5-HT did not change tail SN excitability or SN-MN efficacy in aged II preparations. In mature preparations, 5-HT depolarized tail SNs, increased SN AP duration, and increased excitability during intracellular current injection. 5-HT also increased monosynaptic EPSP amplitude in mature tail MNs. The absence of synaptic facilitation in aged animals may be the result of reduced sensory neuron performance including decreased baseline excitability. Such a sensory deficit would cause the sensitizing training stimulus to be more weakly transmitted in aged animals, thereby interfering with the induction of short-term facilitation and short-term sensitization. Stronger, longer, or more frequent sensitizing stimuli, however, did not induce memory for sensitization or synaptic facilitation in aged animals. When aged SNs were driven to fire the same number of AP as mature in baseline responses, sensitization training failed in aged SNs. These results suggest that impaired sensory input represents only part of the decline in synaptic facilitation in aged animals.

The result that 5-HT was ineffective at inducing synaptic facilitation in aged neurons suggests that the 5-HT receptor-initiated signaling cascade is compromised during aging. During synaptic facilitation, 5-HT released at presynaptic terminals activates molecular pathways that include PKA and PKC signaling, resulting in changes in synaptic plasticity and learning. In short-term facilitation, 5-HT activates adenylyl cyclase through activation of a G protein, G_s_, causing an increase in intracellular cAMP and activation of PKA [24]. Age-related deficits in any part of this signaling cascade would alter induction of synaptic facilitation. 5-HT (20 μM) perfused directly onto ganglia was ineffective in inducing short-term facilitation, suggesting that low 5-HT was not responsible for aging declines in SN excitability or SN-MN efficacy, but that compromised 5-HT receptor physiology or G-protein coupled signaling may have been. Responsiveness to neuromodulators that act through G-protein coupled signaling, including to 5-HT, has been previously shown to decrease with advanced age in several animal models [45–51]. Studies have noted a progressive age-related decline in the postsynaptic response to 5-HT in the rodent hippocampus [46]. Our findings that aged tail SNs did not depolarize or display increased AP duration after 5-HT suggest that components of this signaling pathway deteriorated in aging and that 5-HT receptor performance or immediate downstream components were compromised in aged tail SNs.

Aging-related defects in synaptic facilitation could involve altered G_s_-5-HT-receptor coupling, or failure to activate sufficient adenylyl cyclase, leading to insufficient cAMP or poorly activated PKA. Biochemical studies in rodent hippocampus have described defective cAMP-PKA-dependent signaling in aging [52–54]. Furthermore, stimulation of hippocampal cAMP-PKA signaling has been shown to reverse age-related memory loss and physiological impairments [55]. Thus poor induction of short-term sensitization and synaptic plasticity observed in aged *Aplysia* may reflect modifications in the G_s_-adenylyl cyclase-cAMP-PKA-dependent second messenger cascade. Modifications in other molecular pathways, including PKC signaling, also likely contribute to the age-related reduction in synaptic facilitation.

An additional source of aging declines pertinent to SN-MN physiology is glutamate receptors. Recent studies in vertebrates have reported the loss of subtypes of the AMPA and NMDA types of glutamate receptors in the brain during aging, as well as a decline in glutamate-mediated excitatory transmission [56–64]. AMPA and NMDA receptors contribute to the induction and expression, respectively, of long-term synaptic plasticity, and age-related loss of functional receptors results in impaired synaptic plasticity and learning deficits [60, 65–66]. In *Aplysia*, glutamatergic responses declined during aging in several SN types including tail SNs [27, 41]. It is likely that changes in glutamate receptor composition contribute to the age-related changes in learning and synaptic facilitation observed in this study.

The *Aplysia* model has direct parallels to vertebrate models of aging in the receptors and second messenger cascades involved in age-related memory loss. The 5-HT receptor that mediates synaptic facilitation in *Aplysia*, 5-HT_apAC1_, is linked to G_s_, whose activation initiates the adenylyl cyclase-cAMP-PKA signaling cascade [67]. 5-HT_apAC1_ is most similar to 5-HT_4_ and 5-HT_7_ receptors in vertebrates [67]. Activation of 5-HT_4_ and 5-HT_7_ receptors and the resultant activation of the G_s_ cascade had therapeutic effects on age-related learning and memory deficits, enhancing the formation of memories [68, 69]. The parallels between *Aplysia* and vertebrate neurophysiologies support the prospect that *Aplysia* aging studies may identify molecular targets with therapeutic potential in age-related memory failure. The connection between behavior and the cellular communication accessible by studies of membrane excitability and receptor physiology is most direct in animal models with simple brains. Our results of failure of synaptic enhancement in the same aged animals that showed behavioral declines demonstrate the *Aplysia* model's use in connecting individual neurons and synapses more directly to behavioral aging than is possible in vertebrate models.

In conclusion, the result that aging disrupted the ability to induce behavioral sensitization in TWR suggests that this simple form of nonassociative learning and short-term forms of memory were compromised in aged *Aplysia*, as in other organisms [66, 70]. Short-term synaptic plasticity in the form of facilitation between tail SNs and MNs decreased during aging, whether the sensitizing stimulus was tail shock or the heterosynaptic modulator 5-HT. These results suggest that the cellular mechanisms involved in the induction and maintenance of synaptic facilitation were compromised during aging. Their failure eliminated a simple form of memory in aged *Aplysia*.
